# Development of a Micro-Gripper Using Piezoelectric Bimorphs

**DOI:** 10.3390/s130505826

**Published:** 2013-05-07

**Authors:** Amr M. El-Sayed, Ahmed Abo-Ismail, Moumen T. El-Melegy, Nur Azah Hamzaid, Noor Azuan Abu Osman

**Affiliations:** 1 Department of Biomedical Engineering, Faculty of Engineering, University of Malaya, Kuala Lumpur 50603, Malaysia; E-Mails: azah.hamzaid@um.edu.my (N.A.H.); azuan@um.edu.my (N.A.A.); 2 Mechanical Engineering Department, Faculty of Engineering, Assiut University, Assiut 71516, Egypt; E-Mail: aboismail@aun.edu.eg; 3 Electrical Engineering Department, Faculty of Engineering, Assiut University, Assiut 71516, Egypt; E-Mail: moumen@aun.edu.eg

**Keywords:** piezoelectric actuator, bimorph

## Abstract

Piezoelectric bimorphs have been used as a micro-gripper in many applications, but the system might be complex and the response performance might not have been fully characterized. In this study the dynamic characteristics of bending piezoelectric bimorphs actuators were theoretically and experimentally investigated for micro-gripping applications in terms of deflection along the length, transient response, and frequency response with varying driving voltages and driving signals. In addition, the implementation of a parallel micro-gripper using bending piezoelectric bimorphs was presented. Both fingers were actuated separately to perform mini object handling. The bending piezoelectric bimorphs were fixed as cantilevers and individually driven using a high voltage amplifier and the bimorph deflection was measured using a non contact proximity sensor attached at the tip of one finger. The micro-gripper could perform precise micro-manipulation tasks and could handle objects down to 50 μm in size. This eliminates the need for external actuator extension of the microgripper as the grasping action was achieved directly with the piezoelectric bimorph, thus minimizing the weight and the complexity of the micro-gripper.

## 1. Introduction

Piezoelectric materials are ideal for systems such as micro-grippers due to their fast reaction time and miniaturization potential [1]. They have been used as sensor and actuator components due to their unique reversible electrical and mechanical properties. Applications of piezoelectric materials range from buzzers to diesel engines, fuel injectors, sonar, ultrasound, and nanopositioners in scanning microscopes [2]. As actuators, piezoelectric materials are increasingly important in the latest positioning technology due to their precise displacement [3] and their several other advantages such as quick response, large generative force, and high electromechanical coupling [4]. Piezoelectric actuators are categorized into two configurations: stack actuators and bending actuators. By stacking the piezoelectric layers on top of one another, the cumulative volume of piezoceramics increases the energy delivered to a load. On the other hand, bending actuators consist of multilayers of piezoceramics with greater length than the stacked type. Those multilayers can either be double mounted or single ended as a cantilever [5]. A special case of multilayer bending actuators is the piezoelectric bimorph actuator, which consists of two layers of piezoelectric material connected over their length surfaces. When electric voltage is applied, one layer extends and the other contracts [6,7]. The resultant bending motion becomes the working principle in micro-mechanical applications [8]. Consequently piezoelectric bimorphs have been involved in areas related to precision position control, loudspeakers, vibration damping, noise control, relays, phonograph pick-up, acoustics, and pressure sensing [9].

An important characteristic of bending piezoelectric bimorphs is that the deflection of the bender's tip is dependent on an alternating driving voltage. Many studies have investigated the behavior of piezoelectric actuators. Other studies performed investigations on the nonlinear behavior of bending piezoelectric bimorphs structures under exposure to high electric fields [10], modeling of asymmetrical bending piezoelectric bimorphs structures and the static behavior of the expected bending moment [11], and analytical description of the bending piezoelectric bimorphs' free tip deflection by matrix calculus [12]. The universal deformation state equations were further extended to trimorph bending structures [13]. The free tip deflection of piezoelectric multilayer beam bending actuators under the influence of an electric load was presented by DeVoe and Pisano [14]. The dynamic behavior of a bimorph bending structure excited to bending vibrations by external harmonic forces, bending moments, pressure loads and electrical driving voltages including a flexible plate attached at the free bender's tip had also been established [15,16] and a system of differential equations describing the dynamics of a bimorph was formulated [17]. These establishments of piezoelectric responses contributed towards its application as a micro-manipulating system.

A single-degree-of-freedom micro-manipulator suitable for space robots applications requires lightweight, simplicity, and immunity from magnetic fields [18]. Space robots most commonly require components that can survive at least the rigors of the space and perform exploration, construction, or other tasks. Smart materials are needed for developing some essential parts in space robots for specific applications. For example, robotic hands are used to contact worksite elements safely, quickly, and accurately without accidentally contacting unintended objects or imparting excessive forces beyond those needed for the task. All these tasks require smart materials with minimal time delay to allow distant humans to effectively command the robot to do useful work [19]. End effectors of space based robots must also be dexterous and precisely manage the position of the grasped object. Therefore, a bending piezoelectric bimorph is an ideal solution for an end-effector that could perform such pick-and-place tasks which is the essence of micro-manipulation [20].

Grasping and moving small objects from one location to another depends on the shape and weight of the object, and whether the object is fragile or firm [21]. The basic operation of the micro-gripper depends on the mechanism of the specific type of actuators employed, such as thermo-piezoelectric actuator [20]. The utilized micro-gripper was developed of two parallel lead zirconate titanate (PZT) layers with a fixed range of displacement. Other micro-manipulation mechanisms were designed to enable the tip of the micro-gripper to move in parallel [22]. Static characteristics and control of the micro-manipulator and variation of deflection with the frequency were also reported [1]. This work aims to extend the investigation on parallel micro-gripping in terms of the effect of the sandwiched supporting layer on deflection of the piezoelectric bimorph actuator and to understand to what extent the rigidity of the bimorph varies due to the brass layer between both piezoceramic layers.

The second aim is in light of the implementation of parallel micro-gripper by utilizing the piezoelectric bimorph itself to grasp soft objects instead of attaching additional flexible cantilever [20,21]. It has been well reported that piezoelectric was used as an actuator for driving micro-manipulation of micro-objects [1,22,23]. A piezo-actuator was also utilized as a driver to provide movement to the flexible amplification mechanism of the micro-gripper. It may deliver a large force, but the size is big and it has a complex structure. This study aims to achieve the grasping action directly from the piezoelectric bimorph in order to minimize the weight and complexity of the micro-gripper. In addition, the essential role of the supporting brass layer in providing the essential behavior of the micro-gripper as well as increasing its life cycle was to be established.

This article presents: (i) the basics of a piezoelectric bimorph and the equations of deflection for both the non-supporting sandwiched layer and the brass supporting layer, (ii) the experimental setup that was used for piezoelectric bimorph characterization and also the overall diagram of the micro-gripper in addition to the tests performed for micro-gripper validation, and (iii) the theoretical and experimental results and discussion of the bimorph and the micro-gripper characteristics.

## 2. Configuration of Bending Piezoelectric Bimorphs

**Figure 1.**
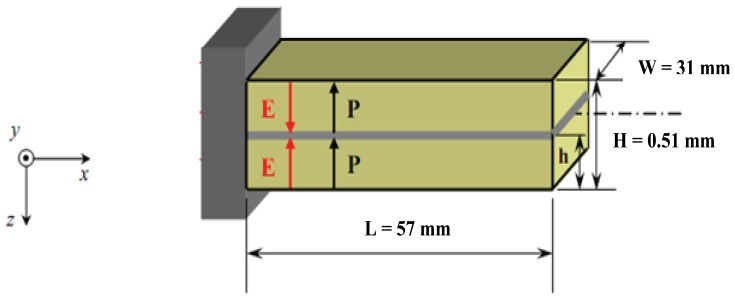
Dimensions, extensive parameters, and polarization of the bimorph actuator.

**Figure 2.**
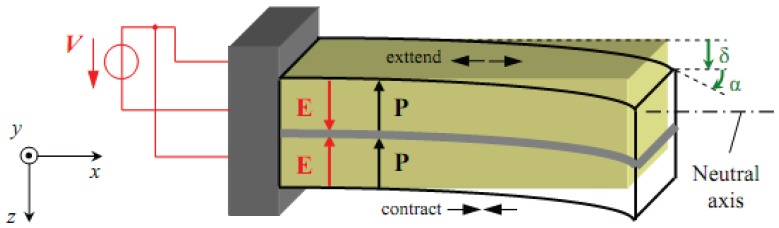
Basic intensive parameters of bimorph actuators after applying electric field.

Bimorphs, which are commonly used as a fundamental element in many operating devices, are made of two piezoelectric sheets bonded together [24]. They were also used to control the vibration of a helicopter rotor blade with limited success [25]. Relations between intensive parameters, which refers to the deflection, bending angle, volume displacement and electrical charge derived for any point over the entire length of the piezoelectric bimorph; and extensive parameters, which refers to variables such as force, bending moment, pressure load and electrical driving voltage [26] have long been established [6,7,10,13,15]. The basic geometry, dimensions, and extensive parameters of our bimorph actuator are shown in Figure 1 where two piezoelectric layers are bonded together with the same polarization. After applying an electric field the piezoelectric bimorph will be deflected as shown in Figure 2.

Generally, piezoelectric bimorph layers are bonded together with a sandwiched supporting material. To establish the relevance of the supporting material, two types of piezoelectric bimorph were employed, in which one of them had the sandwiched supporting material removed. The relationship between the exciting voltage and the output deflection was estimated in both cases [10] and is discussed herein using the equations of deflection *versus* the applied voltage.

Case 1: Without supporting material between the two piezoelectric layers.
  The two layers are identically in geometrical, electrical, and thermal parameters. The analytical bending curvature is given by Equation (1) [10]:
  (1) $$\delta (x)=\frac{3d_{31}x^{2}}{H^{2}}V$$
  where
  *δ (x)*, the deflection at any position -x (mm)
  *d_31_*, piezoelectric coefficient (mm/V)
  *H*, total hickness of piezoelectric actuator (mm)
  *V*, applied voltage (V)
Case 2: With a supporting brass layer between the two piezoelectric layers.

**Figure 3.**
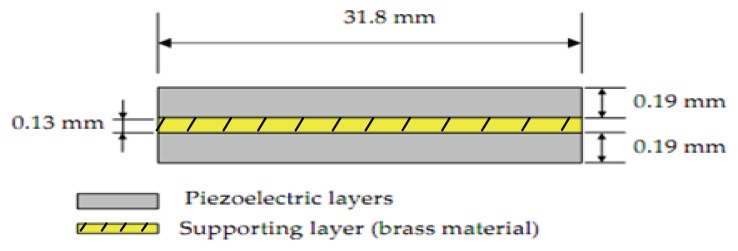
Cross sectional area of the used bimorph actuator.

The typical configuration in this case of bimorph, which consists of a thin brass metal substrate sandwiched between two piezoceramic patches is presented in Figure 3.

Equation (2) [10] shows the constitutive relationship of the triple layer piezoelectric bender with applied electric voltage V and tip deflection δ:

(2) $$\delta =\frac{6s_{11}^{m}d_{31}(h_{m}+h_{p})L^{2}}{2s_{11}^{m}(3h_{m}^{2}h_{p}+6h_{m}h_{p}^{2}+4h_{p}^{3})+s_{11}^{E}h_{m}^{3}}V$$

where

*S^m^_11_*, elastic coefficient of the supporting layer (m^2^/N)
*S^E^_11_*, elastic coefficient of the piezoelectric layer (m^2^/N)
*h_m_*, thickness of the supporting layer (mm)
*h_p_*, thickness of the piezoelectric layer (mm)
*L*, length of the piezoelectric bimorph (mm)

## 3. Experimental Setup

**Figure 4.**
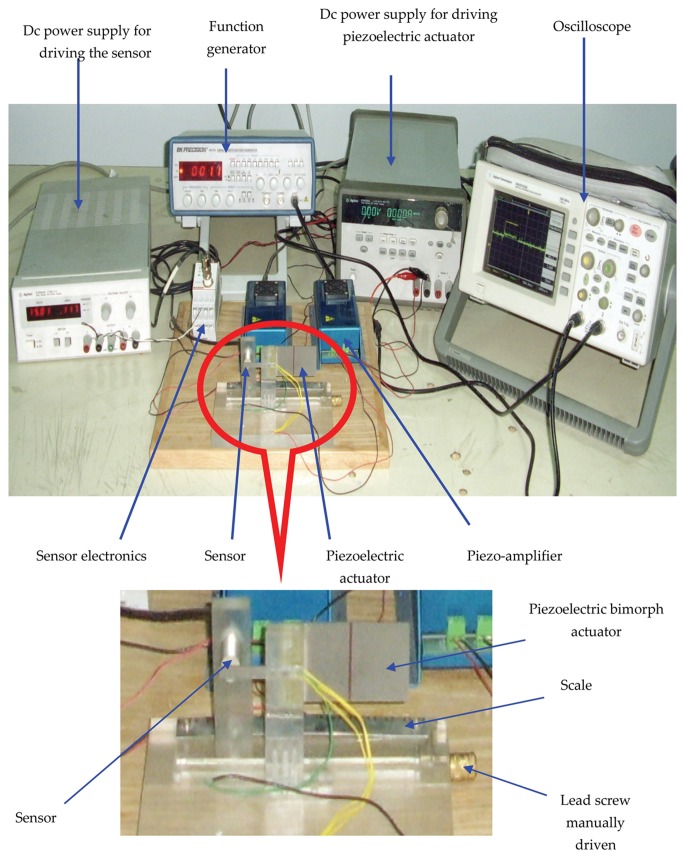
The experimental setup showing the sensor and piezoelectric actuator amongst other components.

**Figure 5.**
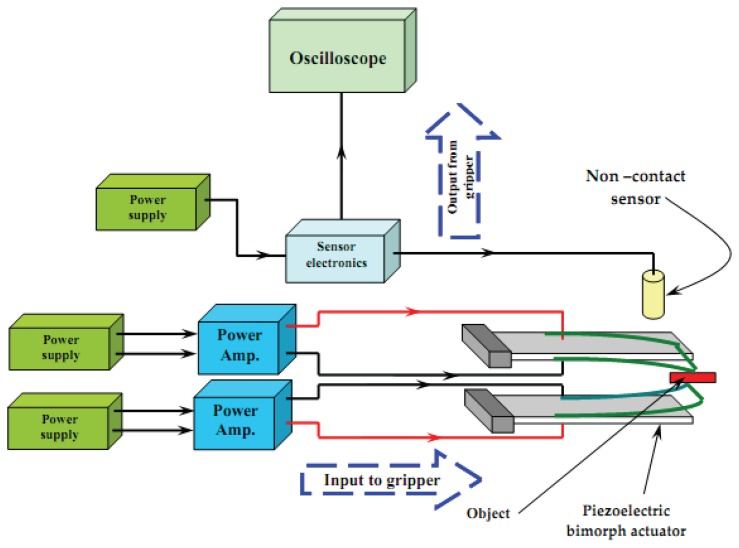
General layout of the developed gripper.

Experimental setup consists of two parts. The first setup was for measurement of static and dynamic characteristics of the piezoelectric bimorph (Figure 4) while the second setup is the general layout of the developed micro-gripper based on the previously obtained characteristics (Figure 5).

### 3.1. Part 1: Static and Dynamic Characteristics Measurement

The apparatus consisted of a bending piezoelectric bimorph of two layers of piezoelectric material bonded together with opposite polarity in the form of a cantilever beam (Figure 4). The bimorph was connected to a mechanical breadboard and driven by a piezo-linear amplifier (Model EPA 007). The EPA-007 was a compact high voltage linear non-inverting amplifier, which was used as a high voltage driving source for the piezoelectric actuating device. The bimorph position was measured using a commercial high-resolution capacitive position sensor mounted on a carriage moved with a lead screw. A DC power supply and function generator were used to generate the drive voltages for the piezo driver. The application of an electric field to the bimorph caused one layer to extend slightly and the other layer to contract slightly in the x-direction. The differential length caused the beam to bend towards the contracting layer. The movement of the cantilever was adjusted by precisely regulating the applied electric field.

### 3.2. Part 2: General Layout of the Developed Micro-Gripper

The developed micro-gripper (Figure 5) consisted mainly of two piezoelectric bending bimorphs, *i.e.*, fingers. Both consisted of two PZT layers in which the bimorph was actuated by applying an electrical voltage across its width. A linear amplifier with a supply input signal drove each finger individually. By applying specific voltage to the bimorph, a definite proportional deflection was produced. The deflection was measured using a non-contact proximity displacement sensor. The output signal from the sensor was displayed on a digital oscilloscope.

**Figure 6.**
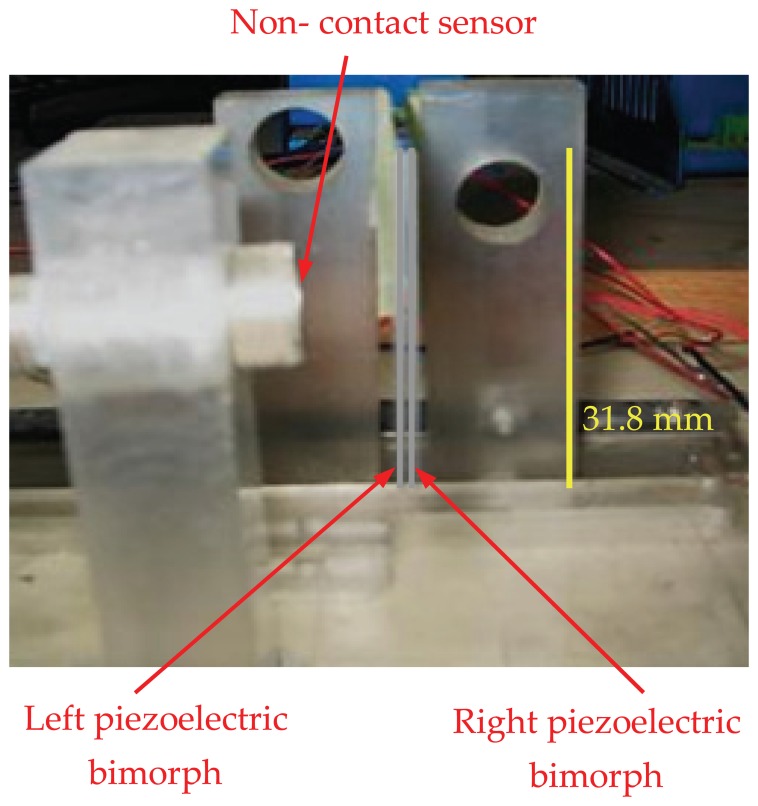
Front view of the two parallel piezoelectric bimorph with the non- contact sensor.

**Figure 7.**
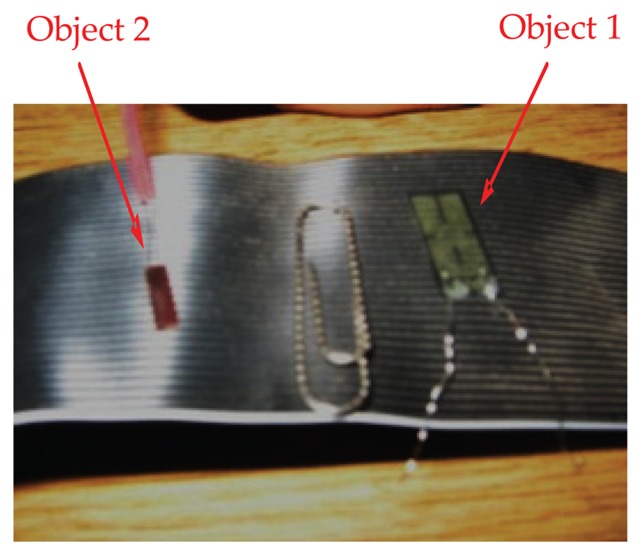
Two types of strain gauges (selected as a micro-objects).

Figure 6 illustrates a two-fingers parallel micro-gripper with a position sensor attached at the tip of the finger. The maximum displacement of the actuator was approximately 2,000 μm. The resulting gripper displacement was sufficient to grasp mini objects. To compensate for the small displacement of the finger, one of the fingers was fixed and the other was mounted on a carriage moved by a lead screw of 1,000 μm resolution. Two different objects were used to test the performance of the micro-gripper. Object 1 was a thin strain gauge (17.5 mm × 7.5 mm × 0.1 mm) and object 2 was a smaller strain gauge (8 mm × 3 mm × 50 μm). Figure 7 shows both objects used for assessment of the micro-gripper performance.

**Figure 8.**
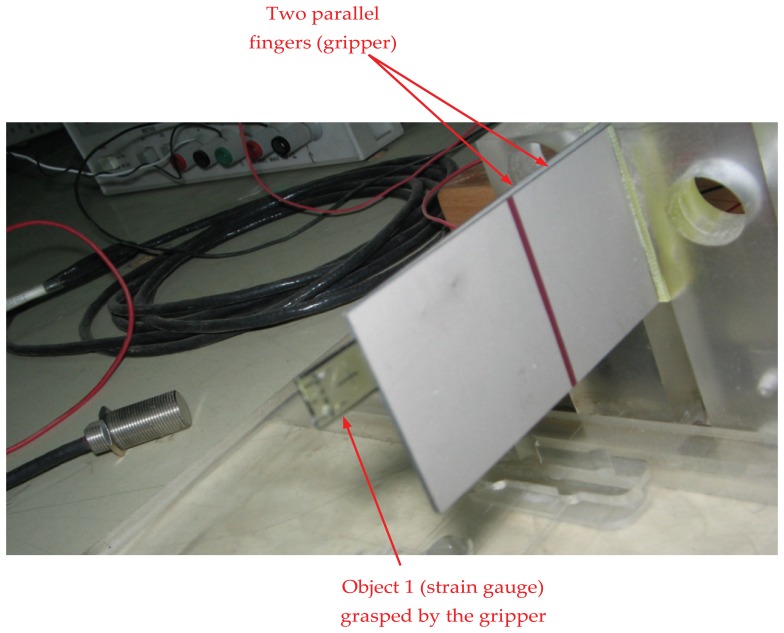
The micro-gripper grasps small object 1 (strain gauge).

**Figure 9.**
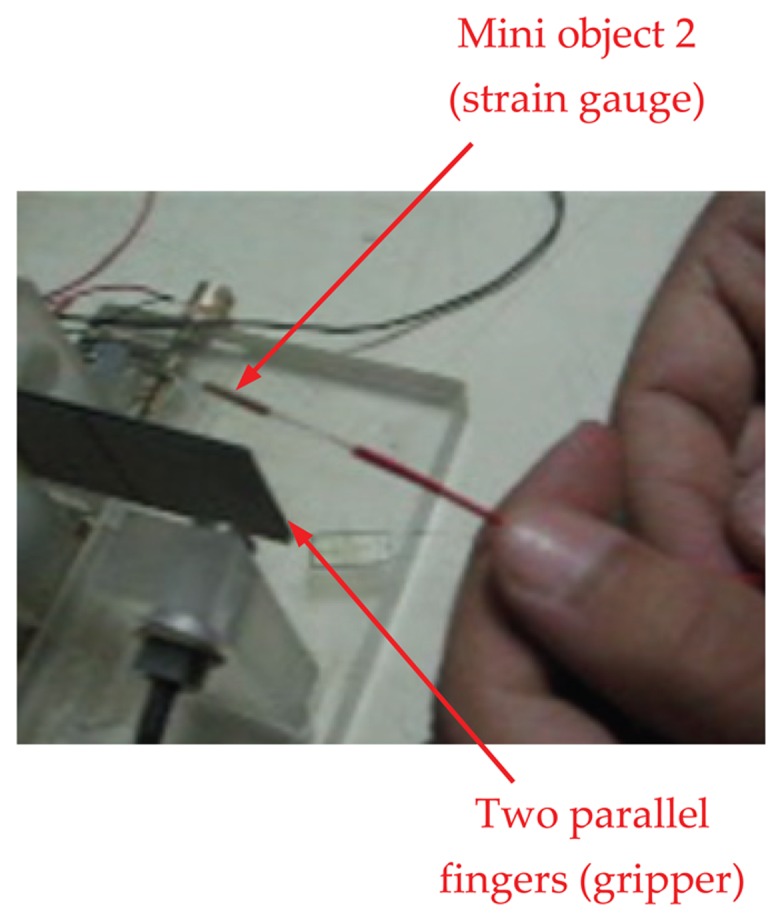
The developed micro-gripper carrying strain gauge (micro-object).

Experiments were performed by grasping object 1, *i.e.*, the strain gauge, as shown in Figure 8. A gap of 100 μm was produced by connecting both fingers with the same voltage. Then, another validation test was performed by picking up object 2 as shown in Figure 9. The micro-gripper successfully grasped the two different objects of different sizes. In the same manner, other objects with various sizes could be manipulated by setting the range of the gap between the two parallel bimorph fingers.

## 4. Results and Discussion

**Figure 10.**
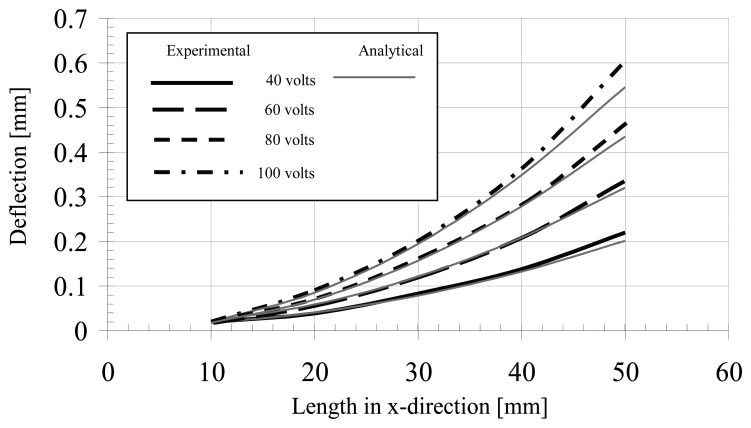
Experimental and analytical deflection of the bimorph according to different applied voltages.

The characteristics of the developed micro-gripper in terms of all possible responses of the piezoelectric bimorphs were presented by measuring the bimorph's deflection. Variations of the measured deflections of the bimorph actuator along the length of the actuator for different driving voltages are illustrated in Figure 10. The experimental investigation was employed by varying the excitation voltage of the bimorph actuator. A bimorph actuator of 57.2 mm length was used for estimating the overall characteristics. The experimental results are correlated with the analytical results within an acceptable error of approximately 2%.

**Figure 11.**
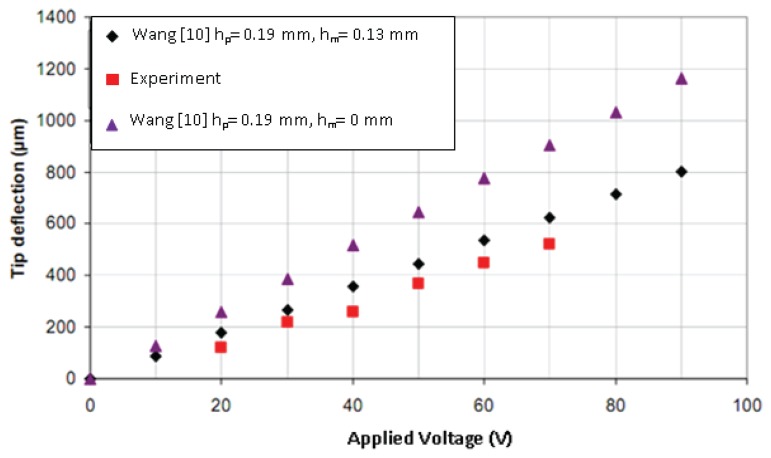
Tip deflection *versus* applied voltage of piezoelectric bimorph, *h_p_*: thickness of the piezoelectric layer (mm), *h_m_*: thickness of the supporting layer (mm).

Further assessment of the tip deflection is presented in Figure 11, which highlighted the performance of experimental result conforming to theoretical values in case 2 where the bimorph is sandwiched with a brass layer of a thickness 0.13 mm.

Nevertheless the results of case 1, *i.e.*, without supporting brass layer, did not agree with the analytical results. This verified the statement regarding the rigidity provided by the supporting layer. From the theoretical calculations of Equation (2) it was confirmed that the bending piezoelectric bimorph without the supporting layer produced a greater tip deflection of up to 1,150 μm, as illustrated in Figure 11.

### 4.1. System Resolution and Sensitivity

The observation of either the force or displacement at the tip of the piezoelectric bimorph might be assessed based on the characteristics of the grasped object. The study assumed that the object to be gripped was delicate and lightweight, thus no slipping occurred during the handling process. Therefore, the assessment was performed for the deflection of piezoelectric bimorph and both resolution and sensitivity were considered to characterize the utilized bimorph.

Resolution of this system was defined as the output displacement of the device corresponding to the input voltage [27–29]. The resolution of the piezoelectric bimorph was dependent on the sensor used to measure the resulting displacement [30]. Results showed the resolution of the utilized piezoelectric bimorph finger for micro-positioning actions to be about 80 μm, based on the non-contact proximity sensor used in the microgripper system. The resolution in terms of grasping action ability, defined by the thickness of the smallest object the gripper can grasp, was demonstrated through the experiment of grasping object 2 in this case, *i.e.*, 50 μm, which was based on the smallest gap between the fingers.

**Figure 12.**
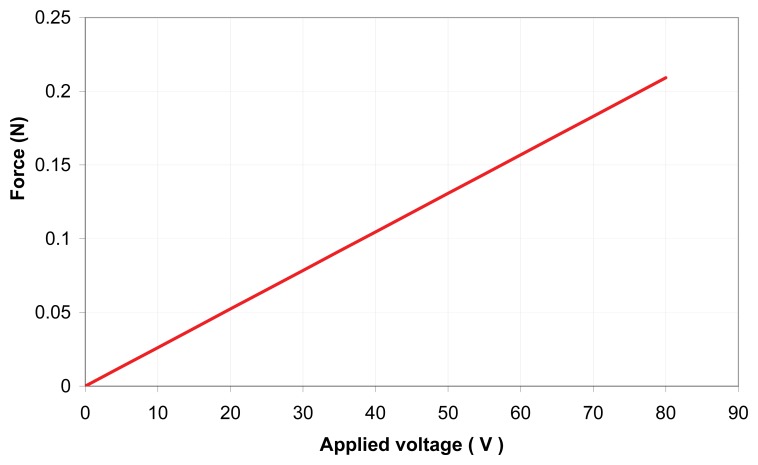
Blocking force at the tip of piezoelectric bimorph *versus* applied voltage.

**Figure 13.**
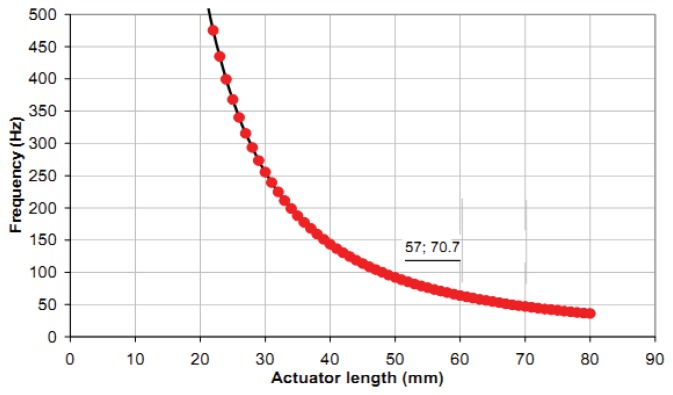
Relationship between frequency and actuator length.

Sensitivity indicates the amount of change in the output, *i.e.*, the displacement of the bimorph, as a result of change in the input, *i.e.*, the excited voltage [27–29]. The sensitivity in the current application was determined from the gradient of the output displacement *versus* input voltage graph (Figure 11) through experimental investigation to be 4 μm/V. The relationship between the input voltage, V, and the blocking force, F, was 2.5 mN/V, derived from a theoretical relationship [10] as shown in Figure 12. The frequency curve of bending piezoelectric bimorph *versus* the length is shown in Figure 13, in which the rate of the natural frequency using bimorph of 57 mm in length is about 70 Hz.

### 4.2. Step Responses

**Figure 14.**
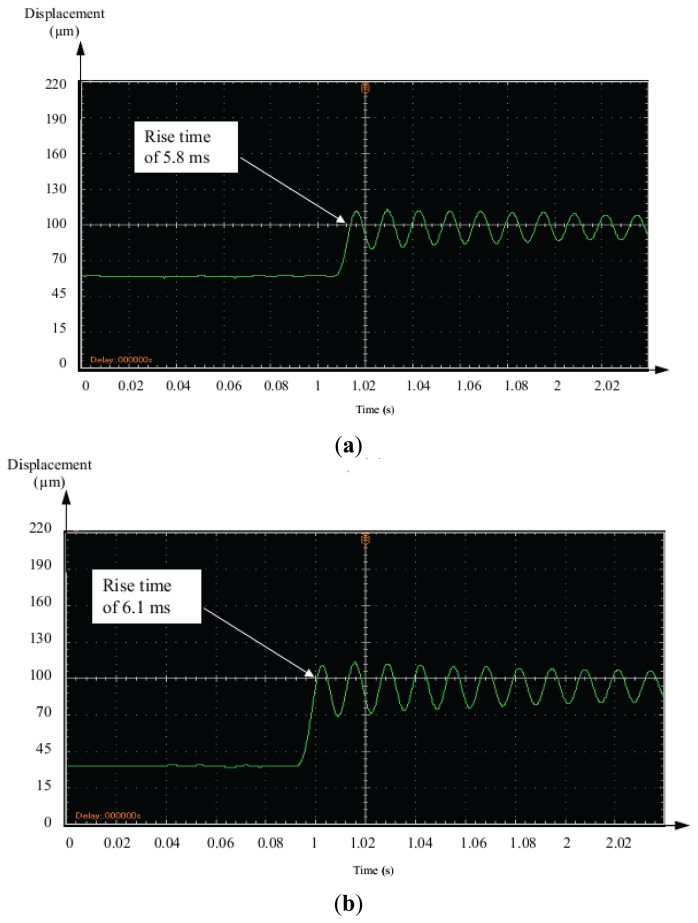
Transient response due to different step voltages. (**a**) 40 μm; (**b**) 70 μm.

The step displacement responses of the piezoelectric bimorph due to different input amplitudes of 40 μm and 70 μm are illustrated in Figure 14, respectively.

The results indicated a fast response time of 0.05 s. However, the rise time was about 5.8 ms and 6.1 ms respectively. This satisfied the micro-gripper specification of control performance.

## 5. Dynamic Response of the Piezoelectric Bimorph

**Figure 15.**
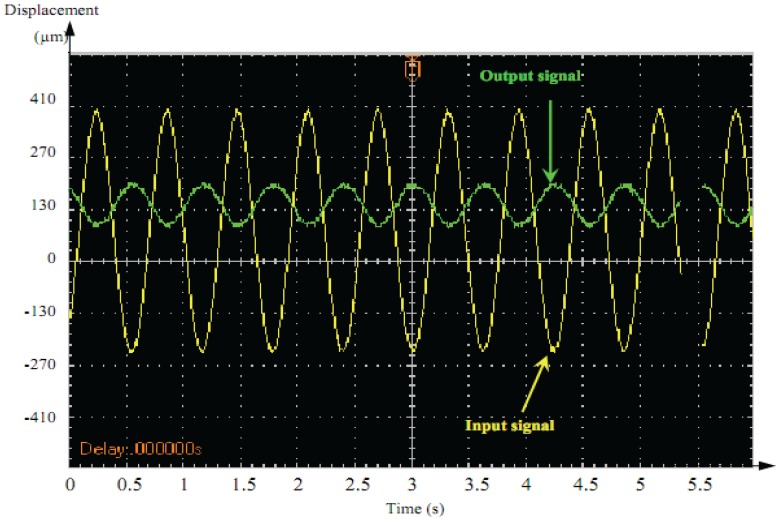
Sine-wave signal.

**Figure 16.**
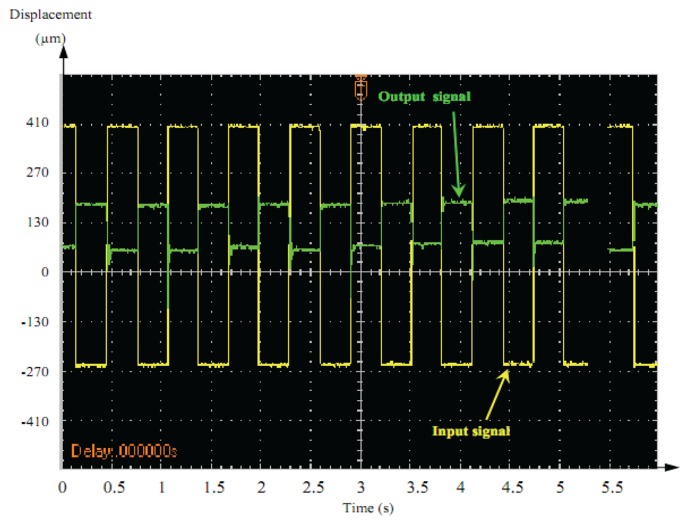
Square-wave signal.

Figures 15 and 16 illustrate the dynamic displacement response under different AC driving signals of the actuator (1 Hz, 20 V).

The output response of the square-wave was characterized by vibration followed by overshoot at the front edge of the square–wave driving voltage. A sine-wave output signal showed an acceptable response compared to square-wave signals. Therefore, sine–wave was better adopted as the control signal for dynamic precision positioning.

## 6. Frequency Response Measurement

**Figure 17.**
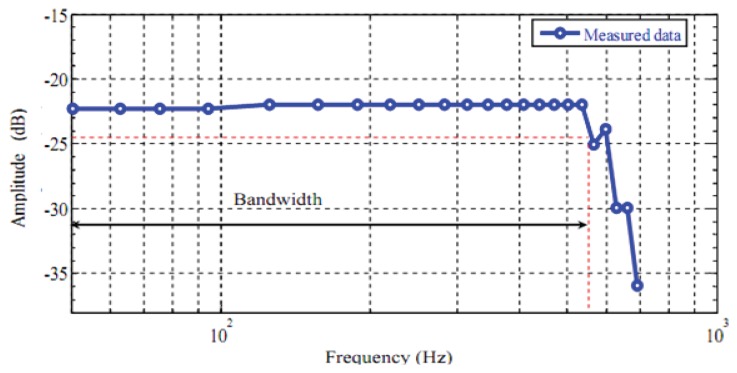
Variation of the deflection with the frequency for bimorph actuator gripper.

The frequency response of the tip displacement of the piezoelectric bimorph was measured with an input signal of 5 V in the frequency range: 0.2–110 Hz and the results are shown in Figure 17.

The bandwidth estimated from the obtained results was 101 Hz for the bimorph actuator. The peaks and valleys show that the piezoelectric bimorph can be described as an underdamped system.

## 7. Conclusions

The characteristics of piezoelectric bimorphs bending actuators were obtained and the experimental setup of the piezoelectric actuator bimorph was successfully developed. The micro-gripper was developed using two parallel piezoelectric bimorphs (fingers) with a non-contact position sensor at the tip of one finger. Each finger was essentially a bending piezoelectric bimorph. To compensate the small displacement of the finger one of the fingers was fixed and the other was supported on a carriage moving on a lead screw, therefore, a sufficient range of object sizes can be handled by changing the initial distance between the fingers. Two micro objects of different dimensions were used as objects to check the validity of the developed micro-gripper. The piezoelectric bimorph micro-gripper time response was reasonable for precise engineering applications with a sine–wave being recommended as the control signal for dynamic precision positioning. This study provided a holistic characterization of a microgripper system for closed loop control as well as the utilization of the piezoelectric material itself as the gripper without requiring additional extension. To measure the micro-gripper displacement and blocking force more advanced sensors with higher accuracy are needed. Further investigations could be undertaken to assess the micro-gripper displacement control and to further develop the micro-gripper to perform more than a single axis movement for advanced automated applications.
